# SERO-prevalence of herpes simplex virus type 1 and type 2 among women attending routine Cervicare clinics in Ghana

**DOI:** 10.1186/s12879-018-3288-1

**Published:** 2018-08-07

**Authors:** Oksana Debrah, Francis Agyemang-Yeboah, Richard Harry Asmah, Emmanuel Timmy-Donkoh, Mohammed Mustapha Seini, Linda Ahenkorah Fondjo, Nilok Sight, Ellis Owusu-Dabo

**Affiliations:** 1 Department of Molecular Medicine, Kwame Nkrumah University of Science and Technology, Kumasi, Ghana; 2 Kumasi Center for Collaborative Research in Tropical Medicine, Kwame Nkrumah University of Science and Technology, Kumasi, Ghana; 3 College of Health Sciences, University of Ghana, Legon, Accra, Ghana; 4 Laboratory Department, Ridge Regional Hospital, Accra, Ghana; 5 Laboratory Department, C&J Medicare Hospital and Diagnostic Center, Accra, Ghana; 6 Department of Global Health, School of Public Health,KNUST, Kumasi, Ghana

**Keywords:** Herpes simplex virus, Sero-prevalence, Ghana

## Abstract

**Background:**

Herpes simplex virus infection is a global health concern with disproportionately high burden in low and middle-income countries. There is a paucity of data on the prevalence of HSV infection in Ghana, which necessitated the present study.

The aim of the study was to provide up-to-date data on sero-prevalence of HSV-1 and HSV-2 infection among women attending Cervicare clinics in Ghana.

**Methods:**

This was a cross-sectional study in which **380** women attending routine Cervicare clinics at Regional Hospitals in Kumasi and Accra, Ghana were enrolled into the study. Serum HSV-1 IgG and HSV-2 IgG were determined by ELISA method. The Chi-square test was used to investigate the association between sero-prevalence of HSV-1 and HSV-2 and socio-demographic and behavioral factors using the Statistical Package for the Social Scientists (SPSS) version 22. Statistical significance was accepted at *p* < 0.05.

**Results:**

The overall HSV-1 and HSV-2 sero-prevalence estimates were 99.2% (95% CI: 98.0–100%) and 78.4% (95% CI: 74.5–81.8%) respectively. The study observed 78.2% cross-positive prevalence of HSV-1 and HSV-2 among the studied participants. There was no association between the presence of HSV-1 and HSV-2 infection and age (χ^2^ = 2.351, *p* = 0.799 and χ^2^ = 1.655, *p* = 0.895 respectively). Our findings however, revealed association between the prevalence of HSV-2 and the age at coitarche (*p* = 0.021) as well as with number of sexual partners (*p* = 0.022).

**Conclusions:**

The sero-prevalence estimates of HSV-1 and HSV-2 among the study population of women in Ghana were found to be high. This high prevalence could be attributed to high endemicity and inadequate intervention in this population. There is the need to raise awareness through organized public health screening and education to ensure control.

**Electronic supplementary material:**

The online version of this article (10.1186/s12879-018-3288-1) contains supplementary material, which is available to authorized users.

## Background

Herpes simplex virus (HSV) has been characterized into two distinct serotypes: HSV -1 and HSV -2 [1]. HSV type 1 has been associated with orofacial infections and HSV type 2 with genital infections. Clinical reports citing an increasing number of genital infections caused by HSV -1 have been recognized, although HSV -2 dominates as a causative agent [1, 2].

Sixty to 95 % of mature humans are either carrying HSV viruses or are affected by associated infections which are usually present in the host in latent state [3]. The large majority of persons with genital herpes do not know they have the disease. Infection and reactivation are typically “asymptomatic,” and depend on the host’s immune system as well as the frequency of entries [3, 4]. Both types are highly infectious and can be transmitted from mother to neonate and increase the mortality rate [5]. Additionally, infection with HSV-2 increases the risk of human immunodeficiency virus (HIV) and human papillomavirus (HPV) acquisition [6, 7]. Estimation of the burden of infection is important in appreciating the scale of the epidemic. Although HSV infection is not a curable medical condition, there are effective medications available to treat symptoms and prevent outbreaks. Unfortunately there is currently no approved vaccine to prevent HSV infection either [8].

The World Health Organization (WHO) reports that HSV prevalence shows variations between regions and populations [9]. The worldwide prevalence of HSV-1 infection in 2012 was 67.0%, with the highest estimated prevalence of infection in Africa (87%) and lowest in America (40–50%). The overall prevalence of HSV -2 worldwide was 11.3% [10]. The prevalence of HSV-2 was consistently higher in females compared to males (14.8 and 8.0% respectively). The highest prevalence was reported in Sub-Saharan Africa, where prevalence reached 31.5% followed by America – 14.4% [9, 10]. In the meantime, there is a paucity of data on the prevalence of HSV infection in Ghana.

The purpose of the study was therefore, to provide relevant baseline data on sero-prevalence of HSV-1 and HSV-2 infection and associated risk factors among women attending routine Cervicare centers in Ghana so as to inform the development of future studies and guide public health policy in the context of HSV infections.

## Methods

### Study design, setting and population

The study was a hospital based cross-sectional descriptive study, covering the period from October 2014 to March 2015.

In all, three hundred and eighty (**380**) women attending routine Cervicare Clinics at the Kumasi South Regional Hospital, Kumasi, Ashanti Region, Ghana and Ridge Regional Hospital, Accra, Greater Accra Region in Ghana were enrolled in the study.

The participants were women who had come to Cervicare centers for visual inspection with acetic acid or to perform Papanicolaou (Pap) smear test. The Cervicare centers were established by Ghana Health Service in selected regional hospitals and health facilities where regular public announcements are carried out to invite the women to participate in screening programs.

### Sampling and data collection

Convenient sampling protocols were followed to recruit the required sample size. The sample size was calculated by StatCalc application of EpiInfo 3.5.3. The approach used here to calculate sample size emphasized adequate precision of reported sample statistics: that is the ability to estimate sample statistics that do not differ from the true population parameter by more than a preset limit of confidence. Therefore, assuming a prevalence of HSV of 67% in the general population of women, and a sufficiently large population of women attending cervical screening clinics, a maximum sample size of 340 women ensured that the study had adequate precision (here we have set the desired level of precision at ±5%) to provide statistics close enough to the true population parameters. The required sample size was pegged at 380 to cater for missing and incomplete data entries and other unforeseen circumstances.

To mitigate bias in the sample, researchers conducted public health awareness campaigns within the catchment of the hospitals: on radio and at market centers and encouraged women to present themselves for screening at no cost. Additionally, a separate day was set aside the regular clinic days to enroll study participants. At recruitment, all volunteers gave informed consent by signature or thumbprint. A questionnaire was administered through one-on-one interview for data collection on socio-demographic and gynecological characteristics, sexual exposure, medical history and knowledge of HSV infection. No participants had symptoms of cervical ulcer from gynecological examination or orofacial ulcer at the time of recruitment.

### Inclusion and exclusion criteria

Participants who were more than 20 years old, non-pregnant and who had written informed consent and had gone through a pre-consented interview were included in the study. Participants, who were less than 20 years old, had previously undergone a cervical examination, were pregnant, had refused to sign an informed consent and were unable to undergo a pre-consented interview were not included in the study.

### Sample collection

Five milliliters (5 ml) of venous blood was drawn from all subjects to determine the presence of HSV-1 IgG and HSV-2 IgG. The samples were allowed to clot before centrifugation. Serum obtained by centrifugation was aliquoted into eppendorf tubes for storage at − 20° C till analyzed.

### Laboratory analysis

The serum HSV-1 IgG and HSV-2 Ig G were determined by ELISA method using commercial test kits from Calbiotech Inc., CA, USA. The manufacturer’s instructions were followed for the analyses. Briefly, 10 μl of serum was diluted with 200 μl of diluent and incubated at room temperature for 5 min. 100 μl of the sample diluent (as a reagent blank), calibrator, negative and positive controls, as well as patients’ serum were then aliquoted into microplate wells in duplicate, and incubated at room temperature for 20 min. Three cycles of washing were performed using 1X washing buffer and 100 μl of anti-IgG conjugate was added and incubated for 20 min. The washing procedure was repeated for another three cycles and 100 μl of substrate solution was added and incubated in the dark at room temperature for 10 min after which the reaction was stopped with 100 μl of stopping solution. The absorbance was measured at 450 nm within 15 min using a reference wavelength of 600 nm – 650 nm. The Antibody (Ab) Index of each determination was calculated by dividing the mean OD value of each sample as well as negative and positive controls by the cut-off value. The cut-off value was calculated as Calibrator OD × Calibrator Factor. Calibrator factor value was indicated on the calibrator bottle.

Wells with patient antibody index greater than 1.1 were conventionally considered positive for the various antibodies tested and those between 0.9 and 1.1 were considered equivocal. While those wells with antibody index less than 0.9 were considered negative for the different antibodies tested. All equivocal samples were retested with reagents of the same kit lot number.

### Statistical analysis

The data collected from the questionnaire responses was stored using Microsoft Excel 2007 software (Microsoft Corporation, Redmond Campus, Washington DC, USA).

Quantitative variables were tested for normal distribution and reported as means ± standard deviation. Qualitative variables were presented as count (percentages). The Chi-square test was used to investigate the association between sero -prevalence of HSV type 1 and type 2, and socio-demographical and behavioral factors using the Statistical Package for the Social Scientists (SPSS) version 22. Statistical significance was conventionally set at *p* < 0.05.

## Results

### Socio-demographic and obstetric characteristics of study participants

The mean age of study participants was 40.83 years (SD ± 11.12) and ranged from 21 to 76 years. Socio-demographic characteristics of study participants are presented in Table 1. The age group from 25 to 44 years was the most represented (63.2%). Majority of the participants were married (58.7%). The literacy rate among the women was very high (91.1%), among which those educated up to the tertiary level were 22.6% (*n* = 86), up to SHS/Vocational- 18.1% (*n* = 69), up to JHS level- 38.7% (*n* = 147) and up to primary- 11.6% (*n* = 44). Majority of women were traders (*n* = 145, 38.2%), self-employed (*n* = 77, 20.3%) followed by government employees (*n* = 51, 13.4%) and private sector employees (*n* = 38, 10.0%).

**Table 1:** Demographics and herpes simplex virus infection sero-prevalence

Characteristics	No tested	Prevalence of HSV infection	
		HSV -1 IgG N (%*)	HSV -2 IgG N (%*)
Age group, years			
≤ 25	12	12 (3.2)	8 (2.1)
25–34	118	116 (30.5)	94 (24.7)
35–44	122	121 (31.8)	94 (24.7)
45–54	80	80 (21.1)	65 (17.1)
55–64	39	39 (10.3)	30 (7.9)
≥ 65	9	9 (2.4)	7 (1.8)
χ^2^ (df), *p*- value		2.351 (5), 0.799	1.655 (5), 0.895
Education			
Never attended	34	34 (8.9)	30 (7.9)
Primary	44	44 (11.6)	36 (9.5)
Junior High School (JHS)	147	146 (38.4)	128 (33.7)
Senior High School (SHS)	45	45 (11.8)	30 (7.9)
Technical/ vocational	24	24 (6.3)	16 (4.2)
Tertiary	86	84 (22.1)	58 (15.3)
χ^2^ (df), *p*- value		3.783 (5), 0.581	20.500 (5), 0.001
Marital status			
Never married	61	59 (15.5)	45 (11.8)
Divorced/separated	33	33 (8.7)	29 (7.6)
Married	223	222 (58.4)	174 (45.8)
Cohabiting	31	31 (8.2)	23 (6.1)
Widowed	32	32 (8.4)	27 (7.1)
χ^2^ (df), p- value		5.921 (4), 0.205	3.542 (4), 0.472
Occupation			
Government employee	51	50 (13.2)	39 (10.3)
Private sector employee	38	37 (9.7)	30 (7.9)
Self -employed	77	76 (20.0)	50 (13.2)
Trader	145	145 (38.2)	128 (33.7)
Subsistence farming	4	4 (1.1)	4 (1.1)
Student	12	12 (3.2)	8 (2.1)
House wife	5	5 (1.3)	3 (0.8)
Unemployed	35	35 (9.2)	26 (6.8)
Retired	13	13 (3.4)	10 (2.6)
χ^2^ (df), p- value		4.498 (8), 0.810	20.172 (8), 0.010

*- Column percentages computed in relation to total number of women (380)

While about fifth of participants, 19.2% were nulliparous, a large proportion of them had their first pregnancy between the age of 18–25 years (*n* = 195, 51.3%) (Table 2). The study also sought to determine the level of awareness of women pertaining to HSV and its mode of transmission (Additional file 1). However, although a few (*n* = 37, 9.7%) women had heard about the term Herpes, none of the women knew about HSV or its mode of transmission.

**Table 2:** Obstetric characteristics and herpes simplex virus infection sero-prevalence

Characteristics	No. tested	Prevalence of HSV infection	
		HSV 1 IgG N (%*)	HSV 2 IgG N (%*)
Age at first pregnancy, years			
≤ 17	36	35 (9.2)	29 (7.6)
18–21	98	98 (25.8)	86 (22.6)
22–25	97	96 (25.3)	81 (21.3)
> 25	72	72 (18.9)	53 (13.9)
Never pregnant	45	43 (11.3)	27 (7.1)
Do not remember	32	32 (8.4)	22 (5.8)
χ^2^ (df), p- value		9.641 (5), 0.086	17.840 (5), 0.003
Gravidae			
0	45	43 (11.3)	27 (7.1)
1–2	90	90 (23.7)	72 (18.9)
3–4	133	132 (34.7)	109 (28.7)
≥ 5	112	112 (29.5)	90 (23.7)
χ^2^ (df), p- value		9.285 (3), 0.026	10.386 (3), 0.016

*Column percentages computed in relation to total number of women (380)

### Sero-prevalence of study participants

The overall HSV-1 and HSV-2 sero-prevalence were 99.2% (95% CI: 98.0–100.0%) and 78.4% (95% CI: 74.5–81.8%) respectively (Tables 3 and 4). The majority of seropositive participants for HSV-1 and HSV-2 were between 25 and 44 years (*n* = 237), and the least rates were among those 65 years and older (*n* = 9) (Table 1). Chi-square analysis did not indicate any association between the occurrences of HSV-1 and HSV-2 infection and age groups (χ^2^ = 2.351, *p* = 0.799 and χ^2^ = 1.655, *p* = 0.895 respectively). Overall, sero-prevalence of both types of herpes infection did not differ by marital status of participants (*p* = 0.205 for HSV-1 and *p* = 0.472 for HSV-2). A strong association existed between HSV-2 infection and level of education among participants (*p* = 0.001) and the age of first pregnancy (*p* = 0.003), but not in the case of HSV-1 infection (*p* = 0.581 and *p* = 0.086 respectively).

**Table 3:** Sero-prevalence of HSV-1 IgG and HSV-2 IgG among the study participants

Result	HSV-1 IgG	HSV-2 IgG
	N (%)	N (%)
Positive	377 (99.2)	298 (78.4)
Borderline	1 (0.3)	54 (14.2)
Negative	2 (0.5)	28 (7.4)

**Table 4:** HSV-1 IgG and HSV-2 IgG positivity stratified by ELISA OD index

Result	HSV-1 IgG	HSV-2 IgG
	N (%)	N (%)
Low positive	228 (60.0)	203 (53.4)
High positive	149 (39.2)	95 (25%)

Low positive: 1.1 < OD ≤ 2.0; High positive: OD > 2.0

The study observed that cross-positive prevalence of HSV-1 and HSV-2 of study participants was 78.2% (95% CI: 73.9–81.6%). There was no association between multiple infection and age of participants (χ^2^ = 6.702, *p* = 0.753), as the same in case of marital status (χ^2^ = 13.531, *p* = 0.095). A study also showed a strong association between multiple infection and educational status (*p* = 0.006) and the age of first pregnancy (*p* = 0.001).

### Sexual risk factors

The analysis of some behavioral risk factors associated with HSV-1 and HSV-2 sero-prevalence is presented in Table 5. There were significant differences between number of sexual partners and the prevalence of HSV-2 (*p* = 0.022). A higher proportion of women (57.9%) had the first sexual relationship before age 20. The study showed that the prevalence of HSV-2 decreased as the age at coitarche increased. This association was statistically significant (*p* = 0.021). Multiple infection was associated with age of first sexual debut (*p* = 0.004), but not with multiple sexual partners (*p* = 0.137).

**Table 5:** Study population behavioral factors and herpes simplex virus infection sero-prevalence

Characteristics	No. tested	Prevalence of HSV infection	
		HSV 1 IgG N (%*)	HSV 2 IgG N (%*)
Age of coitarche, years			
≤ 15	28	28 (7.4)	23 (6.1)
16–20	192	192 (50.5)	157 (41.3)
21–25	53	51 (13.4)	38 (10.0)
≥ 26	24	24 (6.3)	13 (3.4)
Do not remember	83	82 (21.6)	67 (17.6)
χ^2^ (df), p- value		8.150 (4), 0.086	11.521 (4), 0.021
Number of life time sex partners			
1	155	154 (40.5)	110 (28.9)
2	91	90 (23.7)	73 (19.2)
3–9	127	126 (33.2)	109 (28.7)
10+	7	7 (1.8)	6 (1.6)
χ^2^ (df), p- value		0.208 (3), 0.976	9.598 (3), 0.022

*Column percentages computed in relation to total number of women (380)

## Discussion

There is a paucity of data on sero-prevalence of HSV infection type 1 and type 2 in Ghana. To our knowledge this is the first study on sero-prevalence of HSV-1 and HSV-2 infection among the women presenting to Cervicare centers in Ghana. The study showed a high prevalence of HSV-1 and HSV-2 among the population of women (99.2 and 78.4% respectively).

Our findings are consistent with those of studies among various populations in several African countries [9]. A study conducted among women in urban Uganda and among pregnant women in Benin city of Nigeria also showed very high prevalence of HSV-1 infection: 98 and 96.6% respectively [11, 12] whereas prevalence of HSV-1 infection among pregnant women in Vanuatu was reported as 100% [13]. In 2012, the WHO 2012 reported a global prevalence of HSV-1 of 68%, with the highest prevalence in Africa (87%) [9].

In the case of HSV-2 infection our findings are corroborated by other studies conducted in Ghana [14, 15]. Those studies focused on smaller numbers of participants. One of the studies showed a sero-prevalence of HSV-2 infection among women attending sexually transmitted disease (STD) clinics in Accra and Kumasi (Ghana) of 71% (*n* = 278) [14]. The other study conducted in Ghana among 91 pregnant women also reported a high prevalence of HSV-2 (68%) which is similar to the current findings [15].

Similar high sero-prevalence rates have been reported in other African countries. Among pregnant women in Cote D’Ivoire − 96.5% [16], women attending STD clinics in Bangui (Central Africa Republic) and Nigeria: 95 and 86.4% respectively [14, 17]. The high prevalence of HSV-2 infection on our study could be due to the high transmission of the virus. The lack of awareness of this viral infection among the population and environmental factors could also be contributory factors.

However, the prevalence of HSV-2 was higher compared to estimates from some African studies as well. The prevalence of infection among women was 58% in Uganda [11], 68% in Zimbabwe [18], 55% in Zambia [19] and 28% in Gambia [20]. A study conducted in Sudan among pregnant women reported a prevalence rate of 34.6% for HSV-2 infection [21] and 20.7% in Tanzania [22]. Two independent studies from Nigeria reported lower sero-prevalence of HSV-2 infection among pregnant women, 44.3% [12] and 47.3% [23] than we found.

HSV-2 prevalence is thought to increase with age as infection is lifelong [10]. However, our study did not reveal a significant correlation between age and HSV-2 infection. This finding was also observed in a study conducted among Sudanese pregnant women [21]. The sexual behavioral factors associated with HSV-2 were young age at sexual debut and multiple sexual partners. Other research supports our findings that earlier age of sexual intercourse is associated with prevalence of HSV-2 infection. Our findings correlated with those reported in Nigeria, India and Zimbabwe [23–25].

Some studies had also indicated that HSV-2 infection is associated with multiple sex partners [24]. In our study this behavioral factor was also significantly associated with HSV-2 prevalence (*p* = 0.022).

However, the presence of HSV-1 infection was not related to sexual behavior in this study. This could be because women in our study were infected early in life and already had antibodies against HSV-1 by the time they became sexually active. This is corroborated by a report from WHO, where in Africa and South-East Asia most HSV-1 infection occurred during first 5 years of life, with no new infections in adulthood [9].

The high estimate of HSV infection highlights the need for development of vaccines and other new HSV prevention strategies [10].

In general, there was very low awareness of the clinical symptoms and mode of transmission of the HSV infection among the women enrolled in the study. Some even believed that, lesions around the mouth were symptoms of malaria. Even though herpes infection could look harmless (since the infected person may be asymptomatic and clueless) it is a lifelong infection which could lead to a “silent” spread in the population, with possible debilitating consequences. This implies that public health concern should be seriously directed to this issue in Ghana. There is a necessity for educational programs and improved strategies in patient care, especially in at-risk populations.

## Limitations

The study design used in this work may limit the generalizability of these findings to the broader population because of variability in risk factor profiles among the general population of women and women with health seeking behavior. Because of this, it is possible that prevalence estimates reported here may be slightly higher than in the general population. Any attempt to generalize these studies beyond this population must be made with caution. Nevertheless, we conducted public health awareness campaigns within the catchment of the hospitals: on radio and at market centers and encouraged women to freely present themselves for screening at no cost. Additionally, it is hoped that fixing a separate day aside the regular clinic days to enroll study participants could arrest and mitigate any bias. Also, because these results are based on cross-sectional data, any causal inference is speculative.

## Conclusion

The prevalence of HSV-1 and HSV-2 among the women attending the Cervicare centers in Accra and Kumasi, the two major cities in Ghana was high. The major factor found to be associated with sero-prevalence of HSV-2 was age at coitarche and number of life time sexual partners. This could be due to the high endemicity and inadequate intervention in this population, the lack of awareness of some viral infections among the population and environmental factors. There is the need to raise awareness through organized public health screening and education to ensure control.

## Additional file

Additional file 1: Questionnaire. (DOCX 19 kb)

## Abbreviations

HIV: Human Immunodeficiency Virus
HPV: Human Papillomavirus
HSV: Herpes Simplex Virus
JHS: Junior High School
Pap: Papanicolaou
SHS: Secondary High School
SPSS: Statistical Package for the Social Scientists
WHO: World Health Organization
