# Supplementary material for: SERO-prevalence of herpes simplex virus type 1 and type 2 among women attending routine Cervicare clinics in Ghana
Source: BMC Infect Dis. 2018 Aug 7;18:378. doi: 10.1186/s12879-018-3288-1 (PMC6081947; doi:10.1186/s12879-018-3288-1)
Supplement: Supplementary file 1 — Questionnaire. (DOCX 19 kb) [file 12879_2018_3288_MOESM1_ESM.docx]

**APPENDIX A**

**QUESTIONNAIRE**

**Title of research study:**

**SERO AND GENOTYPIC PREVALENCE OF HERPES SIMPLEX VIRUS TYPE 1 AND TYPE 2 AMONG HUMAN PAPILLOMAVIRUS- INFECTED POPULATION OF WOMEN IN GHANA**

**PRINCIPLE INVESTIGATOR: OKSANA DEBRAH**

**DEPARTMENT OF MOLECULAR MEDICINE,**

**SCHOOL OF MEDICAL SCIENCE,**

**KWAME NKRUMAH UNIVERSITY OF SCIENCE AND TECHNOLOGY,**

**KUMASI**

**SUPERVISORS:**

**PROF. FRANCIS AGYEMANG-YEBOAH**

**PROF. ELLIS OWUSU-DABO**

**MR. RICHARD H. ASMAH**

**I. Identification Information:**

Date of completion of the questionnaire (dd/mm/yyyy) __ __ / __ __ / __ __ __ __

Name of the interviewer __________________________________________

Consent:

Consent has been read out to participant: [ ] Yes [ ] No

If No, read consent [ ]

Consent has been obtained: [ ] Yes [ ] No

**If No END an interview** [ ]

**II. Demographic Information:**

1. Full name of participant …………………………………………………………………

2. Other name, if any ……………………………………………………………………….

3. ID number ………………………………………….

4. Contact address/ phone …………………………………………………………………

………………………………………………………………………………………………

5. What is your date of birth (dd/mm/yyyy)?

__ __ / __ __ / __ __ __ __ [ ] Don’t know [ ] Refused

6. How old are you?

__ __ Years [ ] Don’t know [ ] Refused

7. What is your marital status?

[ ] Never married [ ] Married [ ] Single [ ] Divorced [ ] Widowed [ ] Don’t know [ ] Refused

8. What is your educational background?

[ ] None [ ] Primary [ ] Middle/JHS [ ] Vocational [ ] Secondary/SHS [ ] Tertiary [ ] Don’t know [ ] Refused

9. Which of the following best describes your main work status over the last 12 months?

[ ] Government employee [ ] Non-Government employee

[ ] Self-Employee [ ] Non-paid [ ] Subsistence farming

[ ] Trading [ ] Student [ ] Homemaker

[ ] Retired [ ] Unemployed (able to work)

[ ] Unemployed (unable to work) [ ] Don’t know [ ] Refused

**III. Physical Examination:**

10. Weight (kg)……………Height (cm) …………BMI …………………..

**IV. Sexual and Reproductive History:**

11. Did you ever experience a menstrual cycle in your life?

[ ] Yes [ ] No [ ] Refused

If No, go to Q 13

12. If ‘Yes’, at what age did you have your first menstrual period?

__ __ years [ ] Don’t know [ ] Refused

13. Have you had menopause (12 consecutive months without menstrual bleeding)?

[ ] Yes [ ] No [ ] Don’t know [ ] Refused

If No, go to Q 14

14. If 'Yes', at what age did you have your last menstrual period?

__ __ years [ ] Don’t know [ ] Refused

15. Have you ever been pregnant?

[ ] Yes [ ] No [ ] Refused

16. If 'Yes', how many pregnancies did you ever have?

[ ] 1 [ ] 2 [ ] 3 [ ] 4 [ ] more than 4 [ ] Refused

17. If 'Yes', how old were you when you had your first pregnancy?

__ __ years [ ] Don’t know [ ] Refused

18. Were all your pregnancies successful?

[ ] Yes [ ] No [ ] Don’t know [ ] Refused

19. If ‘No’, please, specify:

[ ] Still birth [ ] Miscarriage [ ] Premature [ ] Abortion

[ ] Don’t know [ ] Refused

20. Have you ever sexual relations with a man?

[ ] Yes [ ] No [ ] Refused

If No, go to Q 27

21. If 'Yes', throughout your life, how many sexual partners have you had?

[ ] One [ ] Two [ ] 3-9 [ ] 10+

[ ] Don’t know [ ] Refused

22. Number of partners in the last 3 months:

[ ] None [ ] One [ ] Two [ ] 3-9 [ ] 10+

[ ] Don’t know [ ] Refused

23. At what age did you have your first sexual partner?

__ __ years [ ] Don’t know [ ] Refused

24. Do you know how many sexual partners your present partner/husband has had?

[ ] One [ ] Two [ ] 3-9 [ ] 10+

[ ] Don’t know [ ] Refused

25. Do you have unprotected sex in the last 3 months?

[ ] Yes [ ] No [ ] Don’t know [ ] Refused

26. Sexually active years:

[ ] ≤ 4 [ ] 5-7 [ ] 8-11 [ ] 12-18 [ ] 19+

[ ] Don’t know [ ] Refused

**V. Clinical history:**

27. Have you ever have sexually transmitted disease?

[ ] Yes [ ] No [ ] Don’t know [ ] Refused

28. If ‘Yes’, please name them?

[ ] Hepatitis B [ ] Hepatitis C [ ] Herpes virus

[ ] Chlamydia infection [ ] Syphilis [ ] Gonorrhea

[ ] Trichimoniasis [ ] Others, specify ……………………………..

**VI. Tobacco and alcohol use:**

29. Do you smoke?

[ ] Yes [ ] No [ ] Refused

30. If ‘Yes’, which best describe you?

[ ] Never smoked [ ] Only tried smoke once

[ ] Used to smoke, but gave up [ ] Smoke occasionally (sometimes)

[ ] Smoke regularly [ ] Don’t know [ ] Refused

31. If you answered ’Yes’ for any of 2, how many cigarettes per day do you smoke?

__ __ cigarettes [ ] Don’t know [ ] Refused

32. If 'Yes', for how long did you smoke (years)?

__ __ years [ ] Don’t know [ ] Refused

33. Have you ever consumed a drink that contains alcohol such as beer, wine, spirit, bitters, palm wine or akpeteshie?

[ ] Yes [ ] No [ ] Don’t know [ ] Refused

If No, go to Q 33

34. If 'Yes,' do you consume these drinks regularly (1 per month for at least 6 months)?

[ ] Yes [ ] No [ ] Don’t know [ ] Refused

**VII. Contraception:**

35. Have you or your partner ever used any contraceptives?

[ ] Yes [ ] No [ ] Don’t know [ ] Refused

36. If ‘Yes’, what are they?

[ ] Pills [ ] Injections [ ] Implanting [ ] Condom

[ ] Don’t know [ ] Refused

37. If it is pills, which one do you take?

[ ] Ovrette [ ] Lo-femenal [ ] Secure [ ] Microgynon

[ ] Don’t know [ ] Refused

**VIII. General knowledge:**

38. Do you know anything about cervical cancer?

[ ] Yes [ ] No [ ] Refused

39. If ‘Yes’, what do you know about it? …………………………………………………………

………………………………………………………………………………………….

40. Has any of your relatives or friends been diagnosed of cervical cancer?

[ ] Yes [ ] No [ ] Don’t know [ ] Refused

41. Do you know anything about Human Papillomavirus?

[ ] Yes [ ] No [ ] Refused

42. If ‘Yes’, what do you know about how it’s spread from one person to another? ………………………………………………………………………………………….

………………………………………………………………………………………….

43. Do you know anything about Herpes simplex virus?

[ ] Yes [ ] No [ ] Refused

44. If ‘Yes’, what do you know about how it’s spread from one person to another? ………………………………………………………………………………………………..

………………………………………………………………………………………………...

45. Do you know anything about Pap smear test?

[ ] Yes [ ] No [ ] Refused

46. If ‘Yes’, what do you know about it? …………………………………………………………

47. Have you ever done Pap smear test?

[ ] Yes [ ] No [ ] Don’t know [ ] Refused

48. If ‘Yes’, do you know the result of Pap smear test?

[ ] Yes [ ] No [ ] Refused

49. If ’Yes’, was it:

[ ] Normal [ ] Abnormal [ ] Don’t know [ ] Refused
